# The SIB Swiss Institute of Bioinformatics Semantic Web of data

**DOI:** 10.1093/nar/gkad902

**Published:** 2023-10-25

**Authors:** SIB Swiss Institute of Bioinformatics RDF Group Members, Adrian Altenhoff, Amos Bairoch, Parit Bansal, Delphine Baratin, Frederic Bastian, Jerven Bolleman*, Alan Bridge, Frédéric Burdet, Katrin Crameri, Jérôme Dauvillier, Christophe Dessimoz, Sebastien Gehant, Natasha Glover, Kristin Gnodtke, Catherine Hayes, Mark Ibberson, Evgenia Kriventseva, Dmitry Kuznetsov, Lisacek Frédérique, Florence Mehl, Tarcisio Mendes de Farias*, Pierre-André Michel, Sébastien Moretti, Anne Morgat, Sabine Österle, Marco Pagni, Nicole Redaschi, Marc Robinson-Rechavi, Kasun Samarasinghe, Ana-Claudia Sima, Damian Szklarczyk, Orlin Topalov, Vasundra Touré, Deepak Unni, Christian von Mering, Julien Wollbrett, Monique Zahn-Zabal*, Evgeny Zdobnov

## Abstract

The SIB Swiss Institute of Bioinformatics (https://www.sib.swiss/) is a federation of bioinformatics research and service groups. The international life science community in academia and industry has been accessing the freely available databases provided by SIB since its inception in 1998. In this paper we present the 11 databases which currently offer semantically enriched data in accordance with the FAIR principles (Findable, Accessible, Interoperable, Reusable), as well as the Swiss Personalized Health Network initiative (SPHN) which also employs this enrichment. The semantic enrichment facilitates the manipulation of large data sets from public databases and private data sets. Examples are provided to illustrate that the data from the SIB databases can not only be queried using precise criteria individually, but also across multiple databases, including a variety of non-SIB databases. Data manipulation, be it exploration, extraction, annotation, combination, and publication, is possible using the SPARQL query language. Providing documentation, tutorials and sample queries makes it easier to navigate this web of semantic data. Through this paper, the reader will discover how the existing SIB knowledge graphs can be leveraged to tackle the complex biological or clinical questions that are being addressed today.

## Graphical Abstract

**Graphical Abstract:**
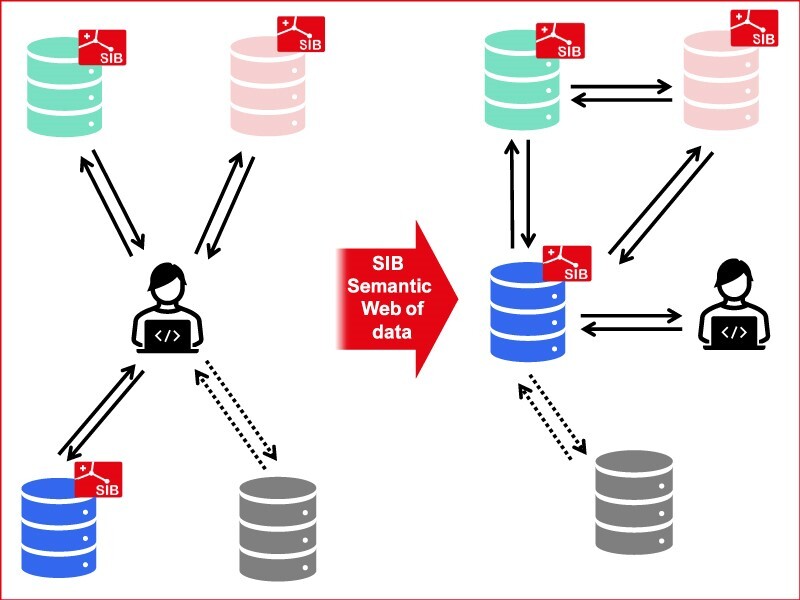

## Introduction

The rapid increase in scientific publications led to literature reviews written by experts in the field and, more recently, to the development of expert-curated databases. The growing production of biological and health data (1) has led to the concomitant growth in the number of databases. While querying is still largely limited to a single database at a time, there is a need to integrate multiple data types (for instance, genomics, transcriptomics, proteomics, metabolomics) to answer complex biological questions. In other words, researchers and health specialists must be able to query and combine data from multiple databases, or even with their own datasets, to gain the insights and knowledge that can only be obtained by seeing the big picture.

The SIB Swiss Institute of Bioinformatics has been active in meeting the needs of the biodata community in academia, industry, and hospitals. With its expertise in data management, storage, integration and analysis, SIB has been developing databases since its inception in 1998. The data in these resources is quite varied, from proteins (their sequences and functions) in UniProt (2), enzymatic and transport reactions catalysed by proteins in Rhea (3), protein–protein interactions in STRING (4), to gene expression in Bgee (5) and orthologs in OMA (6) and OrthoDB (7). All these databases provide scientists worldwide high-quality data to build upon.

Text indexation has made database contents more accessible, thereby establishing them as cornerstone of basic life sciences and medical research. While this makes databases easy to use by humans, it severely limits the types of questions that can be answered through querying. The advent of the Semantic Web (http://www.w3.org/2001/sw/), a web of linked data, allows both humans and machines to navigate between databases that store information about the same entity. Resource Description Framework (RDF; http://www.w3.org/RDF/) is a W3C core Semantic Web technology that is particularly suited to sharing and linking data worldwide. Data in RDF can be queried, retrieved, and manipulated using the SPARQL query language (http://www.w3.org/TR/rdf-sparql-query/). The RDF data model is a directed graph, which can be represented as a set of statements in the form of triplets, subject-predicate-object. To link data on the Web, RDF requires that each entity must have a globally unique identifier. These identifiers allow everybody to make statements about a given entity and, together with the simple structure of the RDF data model, make it easy to combine statements about entities made by different databases to allow queries across different datasets.

In the present article, we present the SIB databases that are part of the global Semantic Web by providing their data as RDF knowledge graphs accessible through SPARQL endpoints. To illustrate how SPARQL queries can be useful to biologists or bioinformaticians, we present a few examples provided by the resources in their SPARQL endpoints. We then proceed to illustrate the use of Semantic Web technologies to explore, link, share, and reuse data, including in the context of the Swiss Personalized Health Network initiative (SPHN) (8). This use case shows how private clinical data can be accessed for research purposes. Finally, given the need to learn SPARQL syntax, we present the training activities undertaken to date to expand the community of users and conclude with future perspectives.

## SIB linked open data in RDF

In contrast to data warehouse initiatives such as the European Bioinformatics Institute (EBI) RDF platform (9) that integrated data from various EBI databases in a centralized repository, SIB databases generate and provide access to their data in RDF independently in a decentralized way. The protein knowledgebase UniProt started exploring the use RDF as early as 2009 (10) and is the largest of the SIB databases provided in RDF (2). The next SIB database to set up a SPARQL endpoint was neXtProt in 2014 (11). OrthoDB followed suit in 2016 (7), followed by OMA (Orthologous Matrix) (12), Rhea (3), Bgee (13), HAMAP (14), MetaNetX (15), and more recently GlyConnect (16), STRING (4) and SwissLipids (17). The Cellosaurus, a SIB knowledge resource on cell lines, does not have a SPARQL endpoint (https://www.cellosaurus.org/). However, part of its cell line data, as well as part of the Bgee expression data, are available via the Wikidata SPARQL endpoint (18). There are currently 11 SIB databases which provide public, linked open data, ranging in topics from proteins, reactions, orthologs, gene expression and metabolomics (Table 1). The SPARQL endpoints listed in Table 1 are all freely available to all via the web, do not require any login or registration, and are not password-protected.

**Table 1.** SIB databases providing free, linked open data for reuse

Database	SPARQL endpoint URL	Type of data
Bgee	https://www.bgee.org/sparql/ https://purl.org/bioquery (Bio-Query)	Gene expression
Cellosaurus	https://query.wikidata.org/ (via Wikidata)	Cell line
GlyConnect	https://beta.glyconnect.expasy.org/sparqlsweets https://glyconnect.expasy.org/sparql (only machine-readable)	Glycoprotein
HAMAP	https://hamap.expasy.org/sparql	Protein family classification and annotation rules
MetaNetX	https://rdf.metanetx.org/	Metabolic network
OMA	https://sparql.omabrowser.org/	Orthologous protein-coding gene
OrthoDB	https://sparql.orthodb.org/	Orthologous protein-coding gene
Rhea	https://sparql.rhea-db.org/	Enzymatic and transport reaction
STRING	https://sparql.string-db.org/	Protein-protein interactions
SwissLipids	https://beta.sparql.swisslipids.org/	Lipid
UniProtKB	https://sparql.uniprot.org/	Protein

Although the SIB RDF resources were created separately and are independently maintained, these resources often reuse data representations, common ontologies, data modelling practices and design patterns from each other to structure their data. This is done to enhance interoperability among SIB resources and to facilitate the writing of SPARQL queries. For example, Bgee and OMA reuse UniProt's data schema and data values (e.g. species) to represent organismal taxonomy. OrthoDB also define organismal taxonomy with UniProt instances. Bgee reuses gene representations from OMA and part of its underlined data schema, Orthology Ontology (ORTH) (12). Moreover, domain specific ontologies such as Gene Ontology (GO) (19) and UBERON (20) (i.e. a multi-species anatomical entity ontology) are integrated within SIB resources, when applicable. For instance, Bgee reuses UBERON; UniProt and OrthoDB reuses GO; MetaNetX and UniProt reuses the ChEBI (Chemical Entities of Biological Interest) ontology (21). In addition, cross-references among SIB and other databases are also modelled with RDF by all SIB resources. For instance, OMA, Bgee and OrthoDB proteins or genes refer to UniProt proteins, facilitating the writing of federated queries to combine their data. Finally, links in RDF go beyond just being a cross-reference, for example, Rhea is used in UniProt to model the catalytic activity of enzymes. This use of Rhea is more than just a pointer, it is indeed a core part of the UniProt data model.

YummyData (22) assesses SPARQL endpoints relevant for biomedical research, as well as the datasets provided, to help users decide which to use and providers to improve the quality of the data provided via Linked Data technologies. The Umaka Score (‘Umaka’ is a Japanese dialect word that means ‘yummy’ in English), is a simple index for quality assessment. YummyData returns scores between 70 and 97 points for the SIB projects, where the maximum score is 100, and the average is 61 (as of 08.2023 - the scores change with time mostly because of ‘data freshness’ criteria). This independent evaluation of the SIB SPARQL endpoints shows their quality and fitness for use.

## Querying RDF data using SPARQL

The SPARQL language allows search criteria to be exquisitely specific. To illustrate this, we present three SPARQL queries that show how life scientists or bioinformaticians can query data in RDF: (i) a query which serves as an example of a search which would not be possible otherwise, (ii) a federated query in which different parts are executed on three different SPARQL endpoints and the retrieved data from them are combined in the result and (iii) a federated query involving two resources, of which one of them is a SIB resource. Importantly, the results of SPARQL queries will always be up to date with the latest information in the SIB resources as the data available in their SPARQL endpoint are updated at each release.

**Figure 1.**
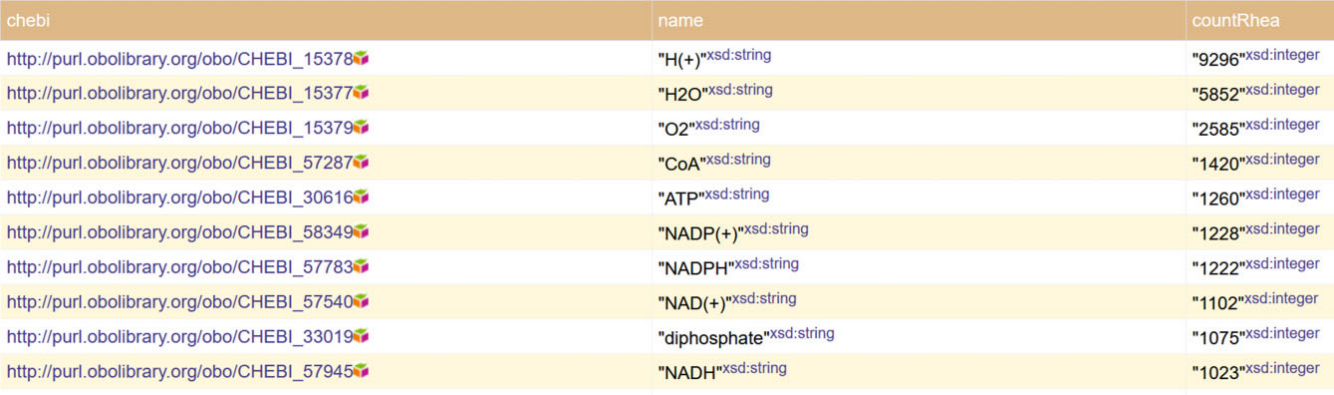
Top ten compounds found in enzymatic and transport reactions found in Rhea and obtained using a SPARQL query. The ChEBI identifier linking to the entry in ChEBI (column chebi), the compound name (column name) and the number of times the compound is found in Rhea (column countRhea) are returned by the query.

As a first example, consider a SPARQL query that cannot be formulated in the text-based search found in the Rhea website. Example 15 in the Rhea SPARQL webpage retrieves all ChEBI compounds used in Rhea as reaction participants, where ChEBI can be either as a small molecule, the reactive part of a macromolecule or as a polymer (the Show Query button displays the SPARQL query, see https://purl.org/sib-rdf/query-example-0001). The ChEBI identifier (linking to its corresponding entry in ChEBI), the compound name and the compound count in Rhea are listed in the results, as illustrated in Figure 1. The results are provided in CSV, XML and JSON format, making it easy to re-use them.

**Figure 2.**
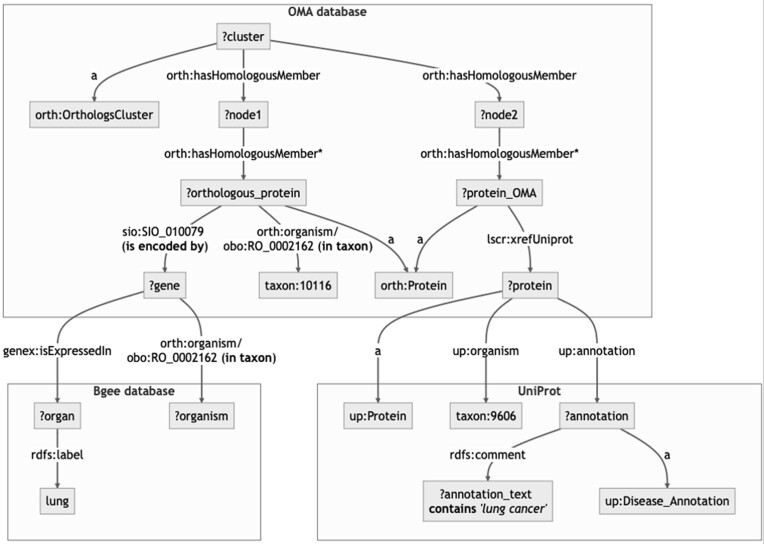
A graphical representation of the semantic query addressed over Bgee, OMA and UniProt databases. This query retrieves the proteins associated with ‘*lung cancer*’ and the orthologs expressed in the rat's *lung*. Nodes with a question mark represent any value of some concept, for instance, *?gene* represents any gene in a given database. Nodes in the form of *prefix:suffix* represents a term in a vocabulary. For example, orth:OrthologousCluster is defined in the ORTHology ontology https://qfo.github.io/OrthologyOntology. Edges in the form of *prefix:suffix* are relations between nodes that are also defined in a vocabulary. For instance, *up:* in *up:annotation* corresponds to *http://purl.uniprot.org/core/*. All prefixes are defined in the header of the SPARQL query. For the sake of simplicity, they were omitted in the figure. Finally, edges with ’*’ means this is a composed edge where the same edge type is repeated as many times as available in the data source. Therefore, it represents the traversal of multiple nodes connected with the same edge type.

Complex biological questions may require different data found in different resources to be queried and combined using a single, federated SPARQL query. All the SIB SPARQL endpoints support the current version of SPARQL (i.e., version 1.1) and thus support federated queries. The Bio-Query interface (https://purl.org/bioquery) is dedicated to federated queries using the data in UniProt, Bgee and OMA. The interface has been designed for users with no knowledge of SPARQL or the underlying data models. Consider a researcher investigating lung cancer who would like to know ‘Which are the proteins associated with ‘*lung cancer*’ and the orthologs expressed in the rat's *lung*?’ To answer this question with Bio-Query, the researcher can edit a question template under the ‘Homologous Genes + Gene Expression + Protein and Functional Information’ category. More precisely, the template question 'Which are the proteins associated with ‘*glioblastoma*’ and the orthologs expressed in the rat's *brain*?' where the researcher should replace *glioblastoma* with *lung cancer* and *brain* with *lung* to compose its original question. This template query illustrates how one can combine the information on homologous genes from OMA with the gene expression data in Bgee and disease annotation in UniProt. The edited template question usually takes less than 10 seconds to return the human UniProt protein links where these links are composed of a UniProt identifier, the OMA link to the corresponding protein expressed in the rat's lung, the OMA gene representation in the RDF graph defined with the Ensembl gene identifier (that is not a clickable link), and the protein disease annotation extracted from UniProt related to lung cancer. Moreover, the federated SPARQL query that is used to answer the edited question can be obtained from the Bio-Query interface by clicking on ‘Show SPARQL Query Editor’ on the top of the page. Alternatively, the SPARQL query can be run on the OMA SPARQL endpoint (see query at https://purl.org/sib-rdf/query-example-0002) or any other SPARQL 1.1. endpoint. Information in a SIB resource can be combined with data found in an external resource. Figure 2 shows a graphical representation of this federated query over those three databases.

**Figure 3.**
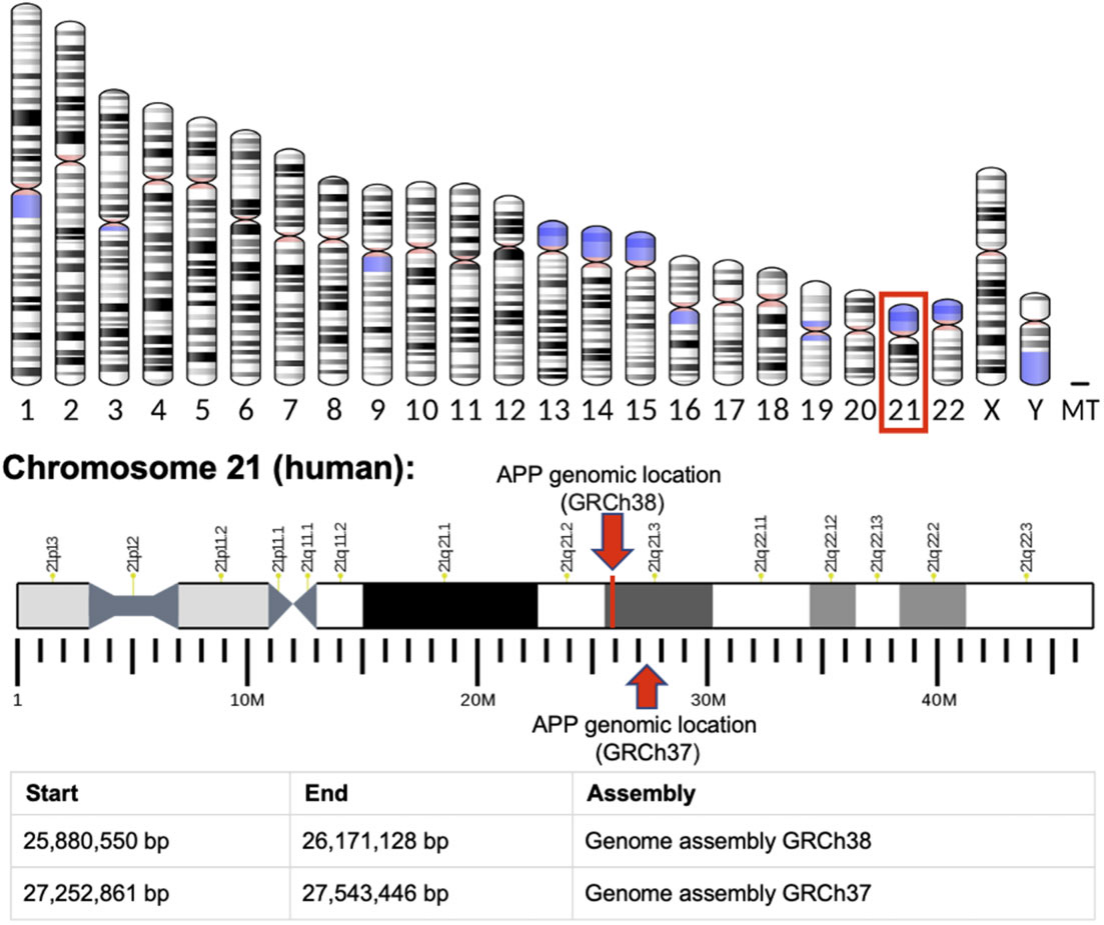
The results of a federated query over Wikidata and UniProt that retrieves the positions of the APP gene in two genome assemblies: GRCh37 and GRCh38. It is known that variants in this gene cause a form of Alzheimer disease.

Another federated query example provided by UniProt (see query 38 at https://purl.org/sib-rdf/query-example-0003) retrieves the positions of the gene start and end in Wikidata for the human entry P05067, amyloid-beta precursor protein (variants in this gene cause a form of Alzheimer disease). The results show that gene coding for the amyloid-beta precursor protein (APP) is found on chromosome 21, extending from positions 25880550–26171128 in genome assembly GRCh38 as depicted in Figure 3. While this information can readily be obtained by searching in either Ensembl or USCS, doing so for a long list of proteins would be tedious; however, the SPARQL query can easily be modified to accommodate a list of protein entries.

The two federated SPARQL query examples illustrate how data silos can be overcome. The selection of SPARQL endpoints shown at https://yummydata.org/endpoint provides additional types of data of interest to life scientists. Of note, Rhea makes use of the Integrated Database of Small Molecules (IDSM) SPARQL endpoint which allows chemical compounds with a similar structure to be retrieved (23). Coudert *et al.* (24) makes use of this functionality to retrieve all proteins that bind to ligands with structures similar to that of a query ligand, in this case, heme b. This type of query could be applied in the context of drug design.

There are several impediments to exploring and using semantic data. The first is becoming familiar with SPARQL syntax. For a programmer or a bioinformatician familiar with a Structured Query Language (SQL), this should not pose any problems. Experimental biologists may learn by running and modifying the examples provided by the resources. The second is an understanding the data model to formulate the queries appropriately. Consulting the documentation or using queries to explore the data will usually overcome this problem. Finally, query timeouts also limit the usefulness of SPARQL querying. This can be overcome by running a query multiple times, each time retrieving a different part of the data.

## Applications of SPARQL and RDF data

The applications of semantic data in RDF and querying with SPARQL are many. They can be used to generate, explore, extract and combine data from various sources, as well as publish data in an interoperable format, to name a few. A few examples are presented below to illustrate some of these uses.

SPARQL not only serves to query data, as illustrated in the previous section – it can be leveraged to annotate data. For instance, Swiss-Prot curators build annotation rules (HAMAP rules), which are used for automatic annotation. HAMAP rules as part of an integrated workflow that includes curation of experimentally characterized template entries in UniProtKB/Swiss-Prot, as well as curation of the associated rule and protein family signature (encoded as a generalized profile). These complex HAMAP rules were translated into the SPARQL 1.1 syntax, and then applied to protein sequences in RDF using freely available SPARQL engines (14). This implementation of HAMAP rules in SPARQL syntax can be applied by users to annotate protein sequences expressed with RDF using off-the-shelf SPARQL engines—without any need for a custom pipeline.

SPARQL queries can also be used to explore and compare data found in different databases. The types of glycans found in glycosylation sites involved in SARS-CoV-2 host-pathogen interactions in GlyConnect and UniProt were recently analysed by combining federated SPARQL queries with manual inspection (25).

Semantic Web technologies can also be used to retrieve data and to combine it with data from a different source, be it public or private, provided reuse is allowed. This enabled the Scalable Precision Medicine Open Knowledge Engine (SPOKE; https://spoke.rbvi.ucsf.edu), to be produced, which contains 27 million nodes and 53 million edges downloaded from 41 databases, including data from Bgee, STRING and UniProt/Swiss-Prot (26). Bgee's high-quality datasets of gene expression were recently integrated into a knowledge graph to enable precision medicine (27). In this way, bridges between data silos are created, and datasets in RDF can readily be disseminated and re-used. Two examples illustrate this. First, a slightly modified version of the neXtProt database was created (https://doi.org/10.5281/zenodo.7071135) and used for a comparison of RDB-to-RDF mapping systems (28). Second, a subset of data in RDF from PDBj (29) has been published in Zenodo (https://doi.org/10.5281/zenodo.8098467) for use in evaluating Oxigraph Server, a graph database implementing the SPARQL standard (30). RDF archives can also serve as a backend for fine-grained version control in collaborative projects.

## Swiss health data in RDF

RDF is also being leveraged in the context of the Swiss Personalized Health Network initiative (SPHN). SPHN has developed a national strategy (8) for the semantic representation of health-related data. At the core of the SPHN Semantic Interoperability Framework is the semantics which is represented formally through SPHN RDF Schema (31). The schema serves as a harmonized model for representing concepts and properties that are relevant for routine clinical data. It is designed in a composable manner and thus offers users the flexibility to extend its capabilities, thereby accommodating their specific requirements. While enabling the seamless integration of diverse data types from heterogeneous sources, the framework also promotes the secondary use of health-data following the FAIR principles.

The developed tools and infrastructures enable Swiss University Hospitals to share clinical routine data defined in the SPHN RDF Schema (https://www.biomedit.ch/rdf/sphn-ontology/sphn) in fast and cost-efficient way. In the current phase of SPHN, four National data streams (NDS) are set up, which link the clinical routine data with other health-related data (e.g. omics data, cohort and registry data) or PROMS in a knowledge graph. The four NDSs focus on different disease area, infectious diseases (Personalized, data-driven prediction and assessment of Infection related outcomes in Swiss ICUs, IICU), oncology (Swiss Personalized Oncology, SPO), low value care (Low Value of Care in Hospitalized Patients, LUCID) and paediatrics (Pediatric personalized research network Switzerland, SwissPedHealth). In the future, the NDS will be an important highly curated data resource for new research projects.

## Documentation and outreach

The majority of SPARQL endpoint users are either programmers, or power users which have invested in learning SPARQL and exploring the resource data models. To lower the barrier for the use of the endpoints by biologists, a user-friendly interface is provided to the SPARQL endpoint for most of the SIB resources. These include SPARQL query examples which allow the naïve user to start by modifying queries, before going on to learning the SPARQL query syntax required to start writing their own queries. Users can also consult the documentation to understand the data model for the resource, retrieve cross-references to SIB or external resources providing additional information, as well as tutorials or training materials (Table 2). In the case of SPHN, training in RDF data, SPARQL and SHACL, as well as a user guide and documentation, are also provided. It should be noted that YummyData (https://yummydata.org/) also provides a forum in GitHub where users and providers of biomedical information in RDF can communicate and improve the usability of the web of (bio) data.

**Table 2.** Documentation, sample queries and training material for SIB databases and SPHN providing semantic data

Database	Documentation	Examples (federated)	Tutorial or training material provided
Bgee	Overview: https://purl.org/sib-rdf/bgee-documentation Data schema: https://purl.org/genex/documentation Query examples: https://purl.org/sib-rdf/bgee-query-examples	19 (14)	http://purl.org/sib-rdf/bgee-tutorial
GlyConnect	−	4 (0)	https://purl.org/sib-rdf/glyconnect-tutorial
HAMAP	−	4 (0)	https://purl.org/sib-rdf/hamap-tutorial
MetaNetX	https://purl.org/sib-rdf/metanetx-documentation	13 (0)	https://purl.org/sib-rdf/metanetx-tutorial
OMA	https://purl.org/sib-rdf/oma-documentation	11 (1)	https://purl.org/sib-rdf/oma-tutorial
OrthoDB	−	17 (1)	https://purl.org/sib-rdf/orthodb-tutorial
Rhea	https://purl.org/sib-rdf/rhea-documentation	17 (3)	https://purl.org/sib-rdf/rhea-tutorial
STRING	https://purl.org/sib-rdf/string-documentation	6 (0)	https://purl.org/sib-rdf/string-tutorial
SwissLipids	−	38 (1)	−
UniProtKB	https://purl.org/sib-rdf/uniprot-documentation	41 (4)	https://purl.org/sib-rdf/uniprot-tutorial
SPHN	https://purl.org/sib-rdf/sphn-documentation		https://purl.org/sib-rdf/sphn-tutorial

Four in-person tutorials have also taken place to date. The first tutorial was an introduction to SPARQL for life scientists at the SWAT4LS 2012 workshop. The second tutorial in 2015 was an introduction to SPARQL for biologists and bioinformaticians at the BC2 conference in Basel. A third tutorial held in 2019 in Edinburgh saw 9 SIB databases presented, with a federated query serving as introduction to the resource presented by the next speaker (slides available at https://purl.org/sib-rdf/2019-swat4hcls-tutorials). The latest tutorials at the SWAT4HCLS 2023 conference in Basel covered UniProtKB, Rhea, as well as SPHN. These tutorials had an indirect impact to foster the collaboration of multiple and independent SIB resources to improve their reusability by enhancing interoperability among them. Moreover, providing tutorials is part of the 10th lesson learned to boost a bioinformatics knowledge base reusability as discussed in (32).

## Concluding remarks

The increasing adoption of Semantic Web technologies to organize biological and biomedical knowledge provides a way to represent the ever-increasing complex interrelationships within and across sub-domains of the life sciences. RDF, a World Wide Web Consortium (W3C) standard, is being used in academy, industry, and governments. It is at the heart of a revolution in which data is not just information but the basis for actionable knowledge. This is urgently needed given the surge in amounts and diversity of data, which are leading to an increase in the number of databases and data repositories. The announcement of funding by the US National Science Foundation to create of a prototype Open Knowledge Network is both timely and needed.

SIB strives to provide a Semantic Web of data across different disciplines in the life sciences. SIB resources contribute high-quality linked data covering a range of topics. This structured data is interlinked with data elsewhere, to be more useful through semantic queries. The federated SPARQL query examples provided in the current SPARQL endpoints interconnect 6 of the 11 SIB SPARQL endpoints, as well as send requests to several external SPARQL endpoints. Future work will focus on identifying and addressing gaps or overlaps between the documentation in a collaborative manner between the projects. A concerted effort is required to add missing equivalences between representations of a same concept by different identifiers in the different databases, as well as strengthen harmonization to further improve their interoperability. The use of standardized metadata across these resources will contribute to the catalogue of machine-readable FAIR datasets. Finally, structuring these data in the form of knowledge graphs enables them to be exploited using artificial intelligence algorithms that offer semantic interpretability and explicability. These algorithms include reasoning based on logical rules extracted from the data, inductive inference based on machine learning of underlying latent relations, and neuro-symbolic combinations of these approaches. These techniques form powerful means of mining, improving, and enriching available knowledge, to help answering complex biological and clinical questions.

## Data Availability

The SPARQL services of the SIB Swiss Institute of Bioinformatics are freely available and listed at https://purl.org/sib-rdf.

## Notes

Full list is provided in Appendix.

## APPENDIX

### SIB Swiss Institute of Bioinformatics RDF Group Members

The following SIB members (a subset of all current SIB members) are co-authors of this article since they participated directly or indirectly in at least one of the resources and/or Swiss bioinformatics activities mentioned in this article: Adrian Altenhoff, Amos Bairoch, Parit Bansal, Delphine Baratin, Frederic Bastian, Jerven Bolleman*, Alan Bridge, Frédéric Burdet, Katrin Crameri, Jérôme Dauvillier, Christophe Dessimoz, Sebastien Gehant, Natasha Glover, Kristin Gnodtke, Catherine Hayes, Mark Ibberson, Evgenia Kriventseva, Dmitry Kuznetsov, Frédérique Lisacek, Florence Mehl, Tarcisio Mendes de Farias*, Pierre-André Michel, Sébastien Moretti, Anne Morgat, Sabine Österle, Marco Pagni, Nicole Redaschi, Marc Robinson-Rechavi, Kasun Samarasinghe, Ana-Claudia Sima, Damian Szklarczyk, Orlin Topalov, Vasundra Touré, Deepak Unni, Christian von Mering, Julien Wollbrett, Monique Zahn-Zabal* and Evgeny Zdobnov.

## References

[1] Holmes D.E. 1. The data explosion. Big Data: A Very Short Introduction. 2017; Oxford University Press 1–13.

[2] The UniProt Consortium Bateman A. , Martin M.-J., Orchard S., Magrane M., Ahmad S., Alpi E., Bowler-Barnett E.H., Britto R., Bye-A-Jee H. et al. UniProt: the Universal Protein Knowledgebase in 2023. Nucleic Acids Res. 2023; 51:D523–D531. 36408920 10.1093/nar/gkac1052 PMC9825514

[3] Lombardot T. , Morgat A., Axelsen K.B., Aimo L., Hyka-Nouspikel N., Niknejad A., Ignatchenko A., Xenarios I., Coudert E., Redaschi N. et al. Updates in Rhea: sPARQLing biochemical reaction data. Nucleic Acids Res. 2019; 47:D596–D600. 30272209 10.1093/nar/gky876 PMC6324061

[4] Szklarczyk D. , Gable A.L., Nastou K.C., Lyon D., Kirsch R., Pyysalo S., Doncheva N.T., Legeay M., Fang T., Bork P. et al. The STRING database in 2021: customizable protein–protein networks, and functional characterization of user-uploaded gene/measurement sets. Nucleic Acids Res. 2021; 49:D605–D612. 33237311 10.1093/nar/gkaa1074 PMC7779004

[5] Bastian F.B. , Roux J., Niknejad A., Comte A., Fonseca Costa S.S., de Farias T.M., Moretti S., Parmentier G., de Laval V.R., Rosikiewicz M. et al. The Bgee suite: integrated curated expression atlas and comparative transcriptomics in animals. Nucleic Acids Res. 2021; 49:D831–D847. 33037820 10.1093/nar/gkaa793 PMC7778977

[6] Altenhoff A.M. , Train C.-M., Gilbert K.J., Mediratta I., Mendes de Farias T., Moi D., Nevers Y., Radoykova H.-S., Rossier V., Warwick Vesztrocy A. et al. OMA orthology in 2021: website overhaul, conserved isoforms, ancestral gene order and more. Nucleic Acids Res. 2021; 49:D373–D379. 33174605 10.1093/nar/gkaa1007 PMC7779010

[7] Zdobnov E.M. , Tegenfeldt F., Kuznetsov D., Waterhouse R.M., Simão F.A., Ioannidis P., Seppey M., Loetscher A., Kriventseva E.V. OrthoDB v9.1: cataloging evolutionary and functional annotations for animal, fungal, plant, archaeal, bacterial and viral orthologs. Nucleic Acids Res. 2017; 45:D744–D749. 27899580 10.1093/nar/gkw1119 PMC5210582

[8] Gaudet-Blavignac C. , Raisaro J.L., Touré V., Österle S., Crameri K., Lovis C. A National, Semantic-Driven, Three-Pillar Strategy to Enable Health Data Secondary Usage Interoperability for Research Within the Swiss Personalized Health Network: methodological Study. JMIR Med. Inform. 2021; 9:e27591. 34185008 10.2196/27591 PMC8277320

[9] Jupp S. , Malone J., Bolleman J., Brandizi M., Davies M., Garcia L., Gaulton A., Gehant S., Laibe C., Redaschi N. et al. The EBI RDF platform: linked open data for the life sciences. Bioinformatics. 2014; 30:1338–1339. 24413672 10.1093/bioinformatics/btt765 PMC3998127

[10] Redaschi N. , Consortium U. UniProt in RDF: tackling Data Integration and Distributed Annotation with the Semantic Web. Nat. Prec. 2009; 10.1038/npre.2009.3193.1.

[11] Gaudet P. , Michel P.-A., Zahn-Zabal M., Cusin I., Duek P.D., Evalet O., Gateau A., Gleizes A., Pereira M., Teixeira D. et al. The neXtProt knowledgebase on human proteins: current status. Nucleic Acids Res. 2015; 43:D764–D770. 25593349 10.1093/nar/gku1178 PMC4383972

[12] de Farias T.M. , Chiba H., Fernández-Breis J.T. Leveraging logical rules for efficacious representation of large orthology datasets. Proceedings of the 10th International Semantic Web Applications and Tools for Healthcare and Life Sciences (SWAT4HCLS) Conference. 2017; 2042:CEUR-WS https://ceur-ws.org/Vol-2042/paper36.pdf.

[13] Sima A.C. , Mendes de Farias T., Zbinden E., Anisimova M., Gil M., Stockinger H., Stockinger K., Robinson-Rechavi M., Dessimoz C. Enabling semantic queries across federated bioinformatics databases. Database. 2019; 2019:baz106. 31697362 10.1093/database/baz106 PMC6836710

[14] Bolleman J. , de Castro E., Baratin D., Gehant S., Cuche B.A., Auchincloss A.H., Coudert E., Hulo C., Masson P., Pedruzzi I. et al. HAMAP as SPARQL rules—A portable annotation pipeline for genomes and proteomes. GigaScience. 2020; 9:giaa003. 32034905 10.1093/gigascience/giaa003 PMC7007698

[15] Moretti S. , Tran V.D.T., Mehl F., Ibberson M., Pagni M. MetaNetX/MNXref: unified namespace for metabolites and biochemical reactions in the context of metabolic models. Nucleic Acids Res. 2021; 49:D570–D574. 33156326 10.1093/nar/gkaa992 PMC7778905

[16] Alocci D. , Mariethoz J., Gastaldello A., Gasteiger E., Karlsson N.G., Kolarich D., Packer N.H., Lisacek F. GlyConnect: glycoproteomics Goes Visual, Interactive, and Analytical. J. Proteome Res. 2019; 18:664–677. 30574787 10.1021/acs.jproteome.8b00766

[17] Aimo L. , Liechti R., Hyka-Nouspikel N., Niknejad A., Gleizes A., Götz L., Kuznetsov D., David F.P.A., Van Der Goot F.G., Riezman H. et al. The SwissLipids knowledgebase for lipid biology. Bioinformatics. 2015; 31:2860–2866. 25943471 10.1093/bioinformatics/btv285 PMC4547616

[18] Waagmeester A. , Stupp G., Burgstaller-Muehlbacher S., Good B.M., Griffith M., Griffith O.L., Hanspers K., Hermjakob H., Hudson T.S., Hybiske K. et al. Wikidata as a knowledge graph for the life sciences. eLife. 2020; 9:e52614. 32180547 10.7554/eLife.52614 PMC7077981

[19] Ashburner M. , Ball C.A., Blake J.A., Botstein D., Butler H., Cherry J.M., Davis A.P., Dolinski K., Dwight S.S., Eppig J.T. et al. Gene Ontology: tool for the unification of biology. Nat. Genet. 2000; 25:25–29. 10802651 10.1038/75556 PMC3037419

[20] Mungall C.J. , Torniai C., Gkoutos G.V., Lewis S.E., Haendel M.A. Uberon, an integrative multi-species anatomy ontology. Genome Biol. 2012; 13:R5. 22293552 10.1186/gb-2012-13-1-r5 PMC3334586

[21] Hastings J. , Owen G., Dekker A., Ennis M., Kale N., Muthukrishnan V., Turner S., Swainston N., Mendes P., Steinbeck C. ChEBI in 2016: improved services and an expanding collection of metabolites. Nucleic Acids Res. 2016; 44:D1214–D1219. 26467479 10.1093/nar/gkv1031 PMC4702775

[22] Yamamoto Y. , Yamaguchi A., Splendiani A. YummyData: providing high-quality open life science data. Database. 2018; 2018:bay022. 29688370 10.1093/database/bay022 PMC5846286

[23] Kratochvíl M. , Vondrášek J., Galgonek J. Interoperable chemical structure search service. J Cheminform. 2019; 11:45. 31254167 10.1186/s13321-019-0367-2 PMC6599361

[24] The UniProt Consortium Coudert E. , Gehant S., de Castro E., Pozzato M., Baratin D., Neto T., Sigrist C.J.A., Redaschi N., Bridge A. et al. Annotation of biologically relevant ligands in UniProtKB using ChEBI. Bioinformatics. 2023; 39:btac793. 36484697 10.1093/bioinformatics/btac793 PMC9825770

[25] Hayes C. , Daponte V., Mariethoz J., Lisacek F. This is GlycoQL. Bioinformatics. 2022; 38:ii162–ii167. 36124803 10.1093/bioinformatics/btac500

[26] Morris J.H. , Soman K., Akbas R.E., Zhou X., Smith B., Meng E.C., Huang C.C., Cerono G., Schenk G., Rizk-Jackson A. et al. The scalable precision medicine open knowledge engine (SPOKE): a massive knowledge graph of biomedical information. Bioinformatics. 2023; 39:btad080. 36759942 10.1093/bioinformatics/btad080 PMC9940622

[27] Chandak P. , Huang K., Zitnik M. Building a knowledge graph to enable precision medicine. Sci. Data. 2023; 10:67. 36732524 10.1038/s41597-023-01960-3 PMC9893183

[28] Galgonek J. , Vondrášek J. A comparison of approaches to accessing existing biological and chemical relational databases via SPARQL. J Cheminform. 2023; 15:61. 37340506 10.1186/s13321-023-00729-5 PMC10280967

[29] Kinjo A.R. , Bekker G.-J., Suzuki H., Tsuchiya Y., Kawabata T., Ikegawa Y., Nakamura H. Protein Data Bank Japan (PDBj): updated user interfaces, resource description framework, analysis tools for large structures. Nucleic Acids Res. 2017; 45:D282–D288. 27789697 10.1093/nar/gkw962 PMC5210648

[30] Yokochi M. , Thalhath N. Evaluating Oxigraph Server as a triple store for small and medium-sized datasets. 2023; BioHackrXiv doi:29 June 2023, pre-print: not peer-reviewed 10.37044/osf.io/yru4b.

[31] Touré V. , Krauss P., Gnodtke K., Buchhorn J., Unni D., Horki P., Raisaro J.L., Kalt K., Teixeira D., Crameri K. et al. FAIRification of health-related data using semantic web technologies in the Swiss Personalized Health Network. Sci. Data. 2023; 10:127. 36899064 10.1038/s41597-023-02028-y PMC10006404

[32] Mendes de Farias T. , Wollbrett J., Robinson-Rechavi M., Bastian F. Lessons learned to boost a bioinformatics knowledge base reusability, the Bgee experience. GigaScience. 2022; 12:giad058. 37589308 10.1093/gigascience/giad058 PMC10433096

